# Transcriptome and membrane fatty acid analyses reveal different strategies for responding to permeating and non-permeating solutes in the bacterium *Sphingomonas wittichii*

**DOI:** 10.1186/1471-2180-11-250

**Published:** 2011-11-14

**Authors:** David R Johnson, Edith Coronado, Silvia K Moreno-Forero, Hermann J Heipieper, Jan Roelof van der Meer

**Affiliations:** 1 Department of Fundamental Microbiology, University of Lausanne, 1015 Lausanne, Switzerland; 2 Department of Environmental Biotechnology, Helmholtz Centre for Environmental Research - UFZ, 04318 Leipzig, Germany; 3 Current Address: Department of Environmental Sciences, Swiss Federal Institute of Technology Zürich (ETHZ), 8092 Zürich, Switzerland; 4 Current Address: Department of Environmental Microbiology, Swiss Federal Institute of Aquatic Science and Technology (Eawag), 8600 Dübendorf, Switzerland

## Abstract

**Background:**

*Sphingomonas wittichii *strain RW1 can completely oxidize dibenzo-*p*-dioxins and dibenzofurans, which are persistent contaminants of soils and sediments. For successful application in soil bioremediation systems, strain RW1 must cope with fluctuations in water availability, or water potential. Thus far, however, little is known about the adaptive strategies used by *Sphingomonas *bacteria to respond to changes in water potential. To improve our understanding, strain RW1 was perturbed with either the cell-permeating solute sodium chloride or the non-permeating solute polyethylene glycol with a molecular weight of 8000 (PEG8000). These solutes are assumed to simulate the solute and matric components of the total water potential, respectively. The responses to these perturbations were then assessed and compared using a combination of growth assays, transcriptome profiling, and membrane fatty acid analyses.

**Results:**

Under conditions producing a similar decrease in water potential but without effect on growth rate, there was only a limited shared response to perturbation with sodium chloride or PEG8000. This shared response included the increased expression of genes involved with trehalose and exopolysaccharide biosynthesis and the reduced expression of genes involved with flagella biosynthesis. Mostly, the responses to perturbation with sodium chloride or PEG8000 were very different. Only sodium chloride triggered the increased expression of two ECF-type RNA polymerase sigma factors and the differential expression of many genes involved with outer membrane and amino acid metabolism. In contrast, only PEG8000 triggered the increased expression of a heat shock-type RNA polymerase sigma factor along with many genes involved with protein turnover and repair. Membrane fatty acid analyses further corroborated these differences. The degree of saturation of membrane fatty acids increased after perturbation with sodium chloride but had the opposite effect and decreased after perturbation with PEG8000.

**Conclusions:**

A combination of growth assays, transcriptome profiling, and membrane fatty acid analyses revealed that permeating and non-permeating solutes trigger different adaptive responses in strain RW1, suggesting these solutes affect cells in fundamentally different ways. Future work is now needed that connects these responses with the responses observed in more realistic scenarios of soil desiccation.

## Background

Dibenzo-*p*-dioxins (DDs) and dibenzofurans (DFs) are widespread and persistent contaminants of soils and sediments and pose a significant threat to human and ecological health. One strategy to mitigate such contamination is to apply bioremediation processes that exploit DD- and DF-degrading members of the *Sphingomonas *group of bacteria [1]. These bacteria use dioxygenase enzyme systems to completely oxidize DD and DF and to co-oxidize many of their chlorinated congeners [2-5]. A previous study with *Sphingomonas wittichii *strain RW1 demonstrated that these enzyme systems are functional when the strain is inoculated into contaminated soils [6], which is promising for bioremediation applications. However, the viability of strain RW1 decreased exponentially after inoculation, with half-lives between 0.9 and 7.5 days [6]. Thus, the soil environment poses significant challenges to the sustained activity and viability of this strain, which could hinder its successful long-term application in bioremediation processes.

Fluctuating water availability, or water potential, is one of the major environmental factors that affect the activity and viability of microorganisms within soils [7-9]. The water potential of a soil is composed of two major components, the solute potential and the matric potential [7,9]. The solute potential is the dominant component in saturated soils and is determined by the concentration and valence state of solutes in solution. A decrease in the solute potential affects the osmotic forces acting on the cell and, unless addressed, can lead to the rapid loss of intracellular water. As an example, the solute potential can dramatically decrease close to the surfaces of plant roots, where the uptake of water by plants can result in an up to 200-fold increase in the concentration of solutes [10]. The matric potential is an important component in unsaturated soils and is determined by interactions between water and solid surfaces [9,11]. A decrease in the matric potential has additional effects on the cell because it reduces the degree of saturation and water connectivity of the soil, which in turn affects the transfer of nutrients and metabolites to and from the cell surface [7].

Microorganisms exploit a number of different adaptive strategies to respond to changes in the water potential, such as accumulating compatible solutes [12] and modifying the compositions of membrane fatty acids [13] and exopolysaccharides [14,15]. In several studies, however, the responses to changes in the solute or matric potential were not identical [13,16]. In those studies, solutes that permeate the cell membrane, such as sodium chloride, were used to control the solute potential while solutes that do not permeate the cell membrane, such as polyethylene glycol with a molecular weight of 8000 (PEG8000), were used to control the matric potential. Because non-permeating solutes reduce the water potential but cannot pass the bacterial membrane, they are often assumed to simulate matric effects in completely mixed and homogeneous systems [8,13,16,17]. Using this approach, a study with *Pseudomonas putida *showed that permeating and non-permeating solutes affected the transcriptional expression of non-identical sets of genes [16]. Other studies with *P. putida *showed that permeating and non-permeating solutes had different effects on the relative amounts of *cis *and *trans *membrane fatty acid isomers [13,18] as well as on the accumulation of the compatible solutes K^+ ^and betaine [19]. Thus, the responses to permeating and non-permeating solutes appear to be different in *P. putida*. Why these responses are different and how these responses are independently regulated, however, is not well understood.

The primary objective of this study was to evaluate how *Sphingomonas wittichii *strain RW1 responds to the permeating solute sodium chloride or the non-permeating solute PEG8000, which are assumed to simulate the solute and matric components of the total water potential, and then compare these responses to identify commonalities and differences between them. The responses of cells were primarily investigated by transcriptome profiling and were further combined with growth rate and membrane fatty acid analyses. The temporal adaptation to these perturbations was also investigated by comparing the short-term and long-term transcriptional responses to sodium chloride and PEG8000. Although other studies have used transcriptome profiling to investigate the responses to changes in water potential [20-25], this study is unique by directly comparing the responses to permeating and non-permeating solutes, which can help reveal whether these solutes affect cells in fundamentally different ways. Moreover, these responses have not been extensively explored in the *Sphingomonas *genus, and this research therefore fills an important gap in our understanding of this bioremediation-relevant group of bacteria.

## Methods

### Growth and culture conditions

*Sphingomonas wittichii *strain RW1 was grown in 100-mL culture flasks containing 20 mL of a phosphate-buffered mineral medium (medium DSM457 from the German Resource Centre for Biological Material, Braunschweig, Germany) and 5 mM of sodium salicylate as the sole carbon source (for simplicity hereafter called DSM457-Sal medium). All cultures were incubated at 30°C with shaking at 180 rpm. The water potential of standard DSM457-Sal medium at 30°C was estimated using the van't Hoff equation [8,11] and is approximately -0.235 MPa.

### Effect of sodium chloride and PEG8000 on the specific growth rate

To investigate the effect of the water potential on the specific growth rate of strain RW1, DSM457-Sal medium was amended with sodium chloride or PEG8000 to reduce the water potential of standard DSM457-Sal medium by 0.25, 0.5, 1.0, 1.5, or 2.5 MPa. The required concentrations of sodium chloride were calculated using the van't Hoff equation [8,11] and were 2.9, 5.8, 11.6, 17.4, or 29 g per L, respectively. The required concentrations of PEG8000 were calculated using a previously described relationship [26] and were 139, 203, 295, 366, and 477 g per L, respectively. Three cultures were prepared for each concentration of sodium chloride or PEG8000 and all cultures were inoculated with a single stationary-phase culture of RW1 (optical density at 600 nM [OD_600_] of 0.8). The growth of strain RW1 was tracked over time by measuring the OD_600 _and zero-order specific growth rates were estimated by linear regression.

### Responses to short-term perturbation with sodium chloride or PEG8000

Precultures containing standard DSM457-Sal medium were inoculated with a single stationary-phase culture of strain RW1 and grown to the mid-exponential phase (OD_600 _of approximately 0.25). Twenty-ml aliquots of preculture were then diluted in triplicate into 180 ml of sodium chloride-amended DSM457-Sal medium (water potential decreased by 0.25 MPa), into 180 ml of PEG8000-amended DSM457-Sal medium (water potential decreased by 0.25 MPa), or into 180 ml of standard DSM457-Sal medium (control cultures). The cultures were then incubated for 30 min, cells were collected by vacuum filtration as described elsewhere [27], and the filters were frozen with liquid nitrogen and stored at -80°C until further processing.

### Responses to long-term perturbation with sodium chloride or PEG8000

Three cultures containing sodium chloride-amended DSM457-Sal medium (water potential decreased by 0.25), three cultures containing PEG8000-amended DSM457-Sal medium (water potential decreased by 0.25 MPa), and three cultures containing standard DSM457-Sal medium (control cultures) were inoculated with a single stationary-phase culture of strain RW1. After inoculation, the cultures were grown for approximately 24 hours until reaching the mid-exponential phase (OD_600 _of approximately 0.25). Cells were then collected by vacuum filtration as described elsewhere [27] and the filters were frozen with liquid nitrogen and stored at -80°C until further processing.

### Microarray design

YODA software [28] was used to design 50-mer probes that target genes from the chromosome and both plasmids of strain RW1. The microarray design has been deposited in the NCBI Gene Expression Omnibus (http://www.ncbi.nlm.nih.gov/geo) under accession number GSE26705 (platform GPL11581) according to MIAME standards [29]. 93% of the probes were designed with the following parameters: 1 to 3 non-overlapping probes per gene, a maximum of 70% identity to non-target sequences, a maximum of 15 consecutive matches to non-target sequences, a melting temperature range of 10°C, and a GC content range of 15%. The remaining 7% of probes were designed with the following less stringent parameters: a maximum of 80% identity to non-target sequences, a melting temperature range of 15°C, and a GC content range of 30%. In total, 12873 probes were designed that target > 99% of the predicted protein coding genes (5323 out of 5345) within the genome of strain RW1. An additional 63 positive and negative control probes were also included in the design. Probes were synthesized on microarrays by Agilent Technologies (Santa Clara, CA) using the 8 × 15,000 format.

### Microarray hybridization and data analysis

RNA was extracted from frozen filters using a previously described acid-phenol method [27,30]. mRNA quality was assessed by verifying intact 16S- and 23S-rRNA bands and by quantifying the A260/A280 and A260/A230 ratios using the MICROARRAY function on a NanoDrop spectrophotometer (ThermoFisher Scientific, Waltham, MA). cDNA was labeled with cyanine-3-labeled dCTP during the reverse transcription step using a modification of a protocol described elsewhere [31]. Briefly, each 50-μl reaction contained 10 μg of total RNA, 1.25 μg of random hexanucleotide primers (Promega, Madison, WI), 100 μM each of unlabeled dATP, dGTP, and dTTP (Life Technologies, Carlsbad, CA), 25 μM of unlabeled dCTP (Life Technologies, Carlsbad, CA), 25 μM of cyanine-3-labeled dCTP (Perkin-Elmer, Waltham, MA), and 400 units of Superscript II reverse transcriptase (Life Technologies, Carlsbad, CA). Reactions were performed by heating at 42°C for 2 hours followed by 70°C for 10 min. RNA was then digested by adding 100 mM of NaOH, heating to 65°C for 20 min, and neutralizing with 100 mM of HCl and 300 mM of sodium acetate (pH 5.2). Labeled cDNA products were purified using the MinElute PCR purification kit (Qiagen, Venlo, Netherlands) and the quantities and incorporation efficiencies of cyanine-3-labeled dCTP were calculated using the MICROARRAY function on a NanoDrop spectrophotometer (ThermoFisher Scientific, Waltham, MA). The incorporation efficiencies typically ranged between 2 and 3%. Sixty ng of labeled cDNA was then loaded onto each microarray, hybridized for 17 hours at 65°C, and washed and scanned as described for labeled cRNA in the One-Color Microarray-Based Gene Expression Analysis Manual (Agilent Technologies, Santa Clara, CA). The fragmentation step (heating to 60°C for 30 minutes) was omitted.

Hybridization signal intensities were extracted from scanned images using the AGILENT FEATURE EXTRACTION software package (version 9.5.3; Agilent Technologies, Santa Clara, CA) and normalized (quantile normalization) and globally scaled using the GENESPRING GX software package (version 10; Agilent Technologies, Santa Clara, CA). All hybridization signals have been deposited in the NCBI Gene Expression Omnibus (http://www.ncbi.nlm.nih.gov/geo) under accession number GSE26705 (samples GSM657248-GSM657272) according to MIAME standards [29]. To test the hypothesis that a gene was differentially expressed between treatment and control conditions, Welch's *t*-test with unequal variances was first used to calculate *p*-values. The Benjamini and Hochberg procedure was then used to correct the *p*-values for multiple hypothesis testing and convert the *p*-values into false discovery rates (FDRs) [32]. For a gene to be classified as differentially expressed between two conditions the FDR had to be less than 0.05 and the fold-difference in hybridization signal intensities had to be greater than two.

### Fatty acid methyl ester (FAME) analysis

Fatty acids were extracted from frozen filters using a chloroform-methanol protocol [33] and converted into fatty acid methyl esters (FAMEs) using a boron trifluoride-methanol protocol [34]. The FAMEs were identified and quantified by gas chromatography-quadrupole mass spectrometry using a protocol described in detail elsewhere [35]. The ratio of saturated to unsaturated fatty acids was quantified as the sum of the relative proportions of palmitic acid (16:0) and stearic acid (18:0) divided by the sum of the relative proportions palmitoleic acid (16:1Δ9*cis*) and *cis*-vaccenic acid (18:1Δ11*cis*), which are the dominant membrane phospholipid fatty acids of this bacterium [36]. The two-tailed Student's *t*-test with a *p*-value cutoff of 0.05 was used to test the hypothesis that the degree of saturation was different between treatment and control cultures.

## Results and discussion

### Sodium chloride and PEG8000 have the same effect on the specific growth rate

**Figure 1:**
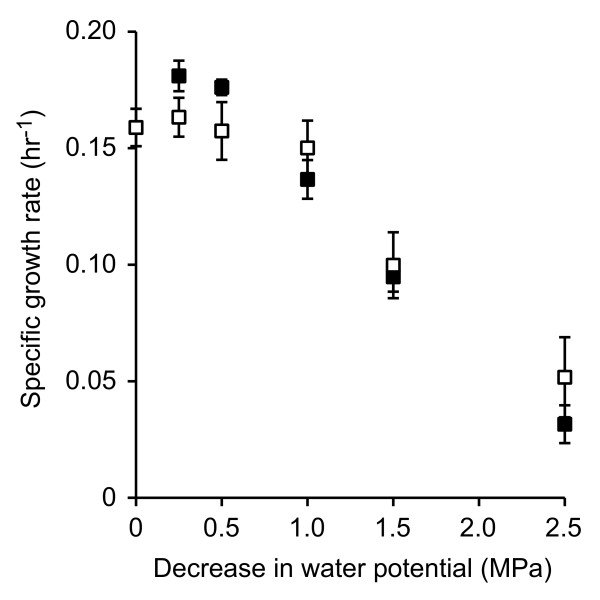
**The effect of sodium chloride or PEG8000 on the specific growth rate of strain RW1**. The water potential was decreased with sodium chloride (filled squares) or PEG8000 (open squares) and zero-order specific growth rates were measured by linear regression. All measurements are averages from three biological cultures and error bars are one standard deviation.

The effect of the permeating solute sodium chloride and the non-permeating solute PEG8000 on the specific growth rate of strain RW1 was tested using liquid batch cultures. A decrease in the water potential by 0.25 to 1.0 MPa with sodium chloride or PEG8000 did not have a substantial effect on the specific growth rate of this strain (Figure 1). A decrease in the water potential by 1.5 MPa, however, significantly reduced the specific growth rate by 37 to 40%, while a further decrease in the water potential by 2.5 MPa reduced the specific growth rate by 67 to 80% (Figure 1). In general, the data indicate that a thermodynamically equivalent decrease in the water potential by adding sodium chloride or PEG8000 had a similar negative effect on the specific growth rate of strain RW1.

### Transcriptional responses to short-term perturbation with sodium chloride or PEG8000

**Figure 2:**
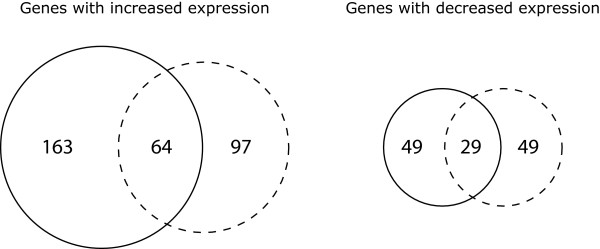
**Summary of genes whose expression levels responded to a short-term perturbation with sodium chloride or PEG8000**. Venn diagrams show the number of genes whose expression levels responded to a short-term perturbation (30 min) with sodium chloride (solid circles) or PEG8000 (dashed circles). The numbers inside the circles indicate the number of differentially expressed genes that had increased or decreased expression (FDR < 0.05, fold difference > 2.0).

Transcriptome profiling was used to identify genes whose expression levels respond to short-term (30 min) perturbation with sodium chloride or PEG8000. A decrease in the water potential by 0.25 MPa was used for transcriptome profiling because this perturbation level did not have a substantial effect on the specific growth rate of strain RW1 (Figure 1). The use of this low level of perturbation reduced the probability of generating non-specific and secondary growth-related effects, and therefore helped to isolate the direct transcriptional responses to these perturbations from the indirect responses that may accumulate when using higher levels of perturbation. Venn diagrams were used to provide an initial overview of the transcriptome data (Figure 2). Overall, a total of 451 genes were differentially expressed after perturbation with sodium chloride or PEG8000, including 93 genes (20.6%) that were differentially expressed by both sodium chloride and PEG8000 (significant differential expression in the same direction) (Figure 2). The direction of differential expression was asymmetrically distributed among the differentially expressed genes, with more genes having increased expression than decreased expression (Figure 2). This was true for perturbation with either sodium chloride or PEG8000.

### Genes whose expression levels responded similarly to a short-term perturbation with sodium chloride or PEG8000

**Figure 3:**
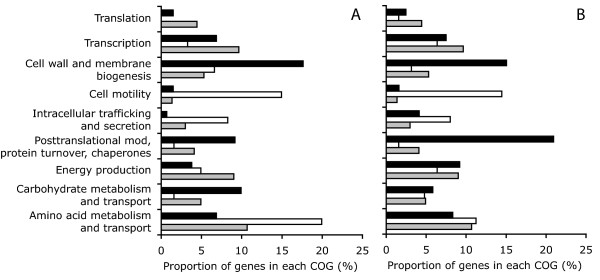
**COG analysis of genes whose expression levels responded to a short-term perturbation with sodium chloride or PEG8000**. The proportion of genes in select cluster of orthologous group (COG) categories were calculated for those whose expression levels were differentially expressed after short-term (30 min) perturbation with sodium chloride (panel A) or PEG8000 (panel B). Proportions were calculated for genes that had increased expression (black bars) or reduced expression (white bars) and were compared to the proportions for all genes within the complete genome (grey bars).

A total of 64 genes had increased expression after short-term perturbation with sodium chloride or PEG8000 (Figure 2 and Additional File 1). These genes include three that are predicted to be sufficient for the complete conversion of glucose-6-phosphate into the compatible solute trehalose (Swit_3608-3610) (Table 1). All three genes are co-localized on the genome and are transcribed in the same direction relative to the origin of replication, suggesting they are likely co-transcribed on a single transcript. None of the other genes in this set are predicted to be involved with the synthesis of other compatible solutes. This leads to the hypothesis that trehalose is a critical compatible solute for adapting to decreasing water potential in strain RW1, which would be consistent with findings made with other environmental microorganisms [9,10,37]. Many genes involved with cell wall and membrane biogenesis also had increased expression after perturbation with chloride or PEG8000 and are over-represented when compared to the complete genome (Figure 3). These include ten genes that are co-localized on the genome and are predicted to encode a pathway for the biosynthesis, export, and assembly of an exopolysaccharide (Swit_4523-4524 and Swit_4526-4533) (Table 1). Exopolysaccharides can act as barriers against the loss of intracellular water to the environment [14,38,39] and microorganisms modify their exopolysaccharide content in response to decreasing water potential [9,14,15]. Another notable gene with increased expression is predicted to encode a rod-shape determining protein (Swit_4023) (Table 1). Homologs of this gene encode a bacterial actin filament that is important for reinforcing the cytoskeletal structure against changes in osmotic forces [40]. Finally, several genes involved with posttranslational modification and protein turnover also had increased expression after perturbation with sodium chloride or PEG8000 and are over-represented when compared to the complete genome (Figure 3). These genes include several heat shock-type chaperones and proteases (Swit_0619, Swit_1146, Swit_1147) (Table 1).

**Table 1:** Select genes whose expression levels responded to short-term (30 min) perturbation with sodium chloride or PEG8000 (FDR < 0.05, fold-difference > 2).

Gene ID	Gene Product	Sodium chloride expression fold-change	PEG8000 expression fold-change	Regulation type
Swit_0619	heat shock protein Hsp20	3.2	6.2	up
Swit_1146	ATP-dependent protease La	3.8	4.8	up
Swit_1147	molecular chaperone (small heat shock protein)-like protein	5.0	3.0	up
Swit_3608	HAD family hydrolase	3.4	2.2	up
Swit_3609	glycoside hydrolase 15-related	8.3	3.9	up
Swit_3610	alpha, alpha-trehalose-phosphate synthase (UDP-forming)	4.0	2.5	up
Swit_4023	rod shape-determining protein MreB	2.3	4.1	up
Swit_4523	glycosyl transferase family protein	4.1	3.8	up
Swit_4524	hypothetical protein	3.3	2.7	up
Swit_4526	glycosyl transferase family protein	2.3	2.8	up
Swit_4527	polysaccharide biosynthesis protein	3.8	3.9	up
Swit_4528	non-specific protein-tyrosine kinase	3.5	3.9	up
Swit_4529	hypothetical protein	2.5	2.4	up
Swit_4530	O-antigen polymerase	3.4	2.9	up
Swit_4531	polysaccharide export protein	4.6	3.1	up
Swit_4532	sugar transferase	16	12	up
Swit_4533	glycoside hydrolase family protein	4.3	3.2	up
Swit_0212	flagellin-specific chaperone FliS-like protein	2.3	2.8	down
Swit_1264	flagellar basal body P-ring protein	2.2	2.3	down
Swit_1267	flagellar basal-body rod protein FlgF	2.2	2.2	down
Swit_1268	flagellar basal body FlaE domain-containing protein	2.4	2.3	down
Swit_1270	flagellar basal-body rod protein FlgC	2.5	2.7	down
Swit_1286	flagellar hook-basal body complex subunit FliE	2.3	2.5	down
Swit_1293	flagellar basal body-associated protein FliL	2.3	2.7	down

An additional 29 genes had reduced expression after short-term perturbation with sodium chloride or PEG8000 (Figure 2 and Additional file 1). These genes are over-represented in genes involved with cell motility when compared to the complete genome (Figure 3) and include seven genes involved with flagella biosynthesis (Swit_0212, Swit_1264, Swit_1267, Swit_1268, Swit_1270, Swit_1286, Swit_1293) (Table 1). The reduced expression of flagella genes in response to decreasing water potential has been reported for a number of different microorganisms [20-23], but the physiological importance of this response remains speculative. The results here suggest that this response is independent of whether the water potential is reduced with permeating or non-permeating solutes.

### Genes whose expression levels responded to a short-term perturbation with sodium chloride but not PEG8000

A total of 163 genes had increased expression after short-term perturbation with sodium chloride but not with PEG8000 (Figure 2 and Additional file 2). These genes include two putative RNA polymerase extracytoplasmic function (ECF) -type sigma 24 factors (Swit_3836, Swit_3924) and adjacent regulatory elements (Swit_3837, Swit_3925, Swit_3926) (Table 2). ECF sigma factors are known to respond to extracytoplasmic signals and to induce the expression of stress response-related genes [41,42]. Thus, these ECF sigma factors might have a role in controlling the response that is specific to sodium chloride. The other genes with increased expression include many with putative roles in the biosynthesis and functioning of the outer membrane (Swit_0142, Swit 0692, Swit_1507, Swit_1509, Swit_2132, Swit_2278, Swit_2322, Swit_3739) and one encoding superoxide dismutase (Swit_2933) (Table 2).

**Table 2:** Select genes whose expression levels responded to short-term (30 min) perturbation with sodium chloride but not PEG8000 (FDR < 0.05, fold-difference > 2.0).

Gene ID	Gene Product	Sodium chloride expression fold-change	Regulation type
Swit_0142	phospholipase D	3.7	Up
Swit_0692	extracellular solute-binding protein	2.8	Up
Swit_1507	17 kDa surface antigen	17	Up
Swit_1509	17 kDa surface antigen	9.3	Up
Swit_2132	peptidoglycan-associated lipoprotein	2.0	up
Swit_2278	OmpA/MotB domain-containing protein	3.6	up
Swit_2322	OmpA/MotB domain-containing protein	10	up
Swit_2933	superoxide dismutase	2.3	up
Swit_3739	chloride channel, core	2.1	up
Swit_3836	ECF subfamily RNA polymerase sigma-24 factor	2.7	up
Swit_3837	putative transmembrane anti-sigma factor	2.5	up
Swit_3924	ECF subfamily RNA polymerase sigma-24 factor	7.2	up
Swit_3925	two-component response regulator	3.5	up
Swit_3926	signal transduction histidine kinase	3.0	up
Swit_0657	glutamate synthase (NADPH) large subunit	2.6	down
Swit_0958	butyryl-CoA:acetate CoA transferase	2.2	down
Swit_0959	3-oxoacid CoA-transferase, A subunit	2.1	down
Swit_2399	methionine synthase (B12-dependent)	2.8	down
Swit_2400	methionine synthase (B12-dependent)	3.0	down
Swit_2401	5,10-methylenetetrahydrofolate reductase	2.8	down
Swit_2559	acyl-CoA synthetase	7.7	down
Swit_2694	glycine cleavage system aminomethyltransferase T	2.0	down
Swit_2696	glycine dehydrogenase subunit 1	2.2	down
Swit_2697	glycine dehydrogenase subunit 2	2.0	down
Swit_3903	diacylglycerol kinase, catalytic region	5.4	down
Swit_3907	fatty acid hydroxylase	3.4	down
Swit_3986	Glu/Leu/Phe/Val dehydrogenase, dimerisation region	2.1	down
Swit_4784	glutamate synthase (NADPH)	2.3	down

An additional 49 genes had reduced expression after short-term perturbation with sodium chloride but not PEG8000 (Figure 2 and Additional file 2). Many of these genes are involved with amino acid metabolism and are over-represented when compared to the complete genome (Figure 3). These include genes involved with the metabolism of glycine (Swit_2694, Swit_2696, Swit_2697), glutamate (Swit_0657, Swit_3986, Swit_4784), and methionine (Swit_2399-2401) (Table 2). Also included were a number of genes involved with lipid metabolism (Swit_0958, Swit_0959, Swit_2559, Swit_3903, Swit_3907) (Table 2).

### Genes whose expression levels responded to a short-term perturbation with PEG8000 but not sodium chloride

A total of 97 genes had increased expression after short-term perturbation with PEG8000 but not with sodium chloride (Figure 2 and Additional file 3). These genes include the RNA polymerase sigma 32 factor (Swit_0060) (Table 3). In other bacteria the sigma 32 factor regulates heat-shock and general stress response systems [43,44]. Consistent with this, genes involved with posttranslational modification, protein turnover, and chaperones were over-represented within this group when compared to the complete genome (Figure 3). These include the chaperones DnaK (Swit_1250) and GroEL (Swit_3376) and other putative genes involved with protein turnover and repair (Swit_0074, Swit_0390, Swit_1939, Swit_2682, Swit_2816, Swit_3375, Swit_3913, Swit_4376, Swit_4377, Swit_4509, Swit_5306, Swit_5351) (Table 3). These results are consistent with a previous study with *P. putida *[16], which also observed the increased expression of a number of chaperones in response to PEG8000 but not to sodium chloride. Although the physiological reason for the increased expression of chaperones only in response to PEG8000 is unclear, these observations suggest that PEG8000 may impact cellular components in a fundamentally different way than sodium chloride.

**Table 3:** Select genes whose expression levels responded to short-term (30 min) perturbation with PEG8000 but not sodium chloride (FDR < 0.05, fold-difference > 2).

Gene ID	Gene Product	PEG8000 expression fold-change	Regulation type
Swit_0060	RNA polymerase factor sigma-32	3.7	up
Swit_0074	peptide methionine sulfoxide reductase	2.3	up
Swit_0390	ATP-dependent protease La	2.4	up
Swit_1250	chaperone protein DnaK	3.6	up
Swit_1939	peptidase M48, Ste24p	3.4	up
Swit_2682	thioredoxin	2.6	up
Swit_2816	methionine-R-sulfoxide reductase	2.5	up
Swit_3375	chaperonin Cpn10	9.5	up
Swit_3376	chaperonin GroEL	9.7	up
Swit_3913	peptidase M23B	2.1	up
Swit_4376	ATP-dependent protease peptidase subunit	3.3	up
Swit_4377	ATP-dependent protease ATP-binding subunit	4.1	up
Swit_4509	membrane protease FtsH catalytic subunit	2.4	up
Swit_5306	heat shock protein DnaJ domain-containing protein	2.2	up
Swit_5351	heat shock protein 90	4.0	up
Swit_2634	benzoate 1,2-dioxygenase, alpha subunit	3.2	down
Swit_3086	gentisate 1 2-dioxygenase-like protein	3.3	down
Swit_3094	glyoxalase/bleomycin resistance protein/dioxygenase	2.8	down
Swit_3864	homogentisate 1,2-dioxygenase	3.6	down
Swit_3865	4-hydroxyphenylpyruvate dioxygenase	3.4	down
Swit_4263	gentisate 1 2-dioxygenase-like protein	2.1	down

An additional 49 genes had reduced expression after short-term perturbation with PEG8000 but not sodium chloride (Figure 2 and Additional file 3). Strikingly, these include six putative dioxygenase-encoding genes (Swit_2634, Swit_3086, Swit_3094, Swit_3864, Swit_3865, Swit_4263) (Table 3). One of these genes is predicted to encode a gentisate 1,2-dioxygenase (Swit_3864) (Table 3), which is involved in the degradation of salicylate in other *Sphingomonas *strains [45].

### Comparison of the short-term and long-term transcriptional responses to sodium chloride and PEG8000

Transcriptome profiling was further used to compare the temporal adaptation to sodium chloride and PEG8000 and to separate the immediate responses from the long-term responses. To achieve this, the responses to short-term perturbation (30 min) with sodium chloride or PEG8000 discussed above were compared with the responses to long-term perturbation (24 hour). For sodium chloride, the expression levels of 305 genes responded to short-term perturbation (Figure 2, Additional file 1 and Additional file 2) while the expression level of only one gene that encodes a hypothetical protein (Swit_0150) responded to long-term perturbation. Thus, the transcriptional state of strain RW1 responded immediately after applying sodium chloride by changing the expression of a large number of genes, but then returned to its initial transcriptional state. A previous transcriptome investigation with *Sinorhizobium meliloti *is consistent with these results. In that study, the number of genes whose expression levels responded to sodium chloride reached a maximum after 30 to 60 minutes and then reduced thereafter [22]. For PEG8000, in contrast, the expression levels of 239 genes responded to short-term perturbation (Figure 2, Additional file 1 and Additional file 3) while of the expression levels of 156 genes responded to long-term perturbation (Additional file 4). Thus, the transcriptional state of strain RW1 changed immediately after applying PEG8000 and remained in a significantly different transcriptional state thereafter.

Of the 156 genes whose expression levels responded to long-term perturbation with PEG8000 (Additional file 4), 19 of the down-regulated genes have predicted functions involved with cell motility, including genes important for the biosynthesis, assembly, and regulation of the flagella (Table 4). These genes are located in three chromosomal regions (Swit_0212-0213, Swit_1260-1293, and Swit_1458) and include a putative Fli-type RNA polymerase sigma-28 factor (Swit_1281), which regulates flagella biosynthesis in other bacteria [46]. Also down-regulated were several genes involved with the biosynthesis and assembly of pili (Swit_0565, Swit_0615, and Swit_0616) (Table 4). In many bacteria flagella and pili have important roles in cell aggregation [47,48]. Consistent with this role, visual and microscopic inspection showed small cell aggregates in control cultures and after long-term perturbation with sodium chloride but not after long-term perturbation with PEG8000 (data not shown).

**Table 4:** Select genes whose expression levels responded to long-term (24 hr) perturbation with PEG8000 (FDR < 0.05, fold-difference > 2).

Gene ID	Gene Product	PEG8000 expression fold-change	Regulation type
Swit_0212	flagellin-specific chaperone FliS-like protein	3.9	down
Swit_0213	flagellar hook-associated 2 domain-containing protein	3.3	down
Swit_0565	type IV pilus assembly PilZ	2.5	down
Swit_0615	Flp/Fap pilin component	2.4	down
Swit_0616	Flp/Fap pilin component	4.9	down
Swit_1260	flagellar motor protein MotA	2.7	down
Swit_1261	flagellin domain-containing protein	2.4	down
Swit_1262	flagellar hook-associated protein FlgK	2.9	down
Swit_1264	flagellar basal body P-ring protein	3.1	down
Swit_1266	flagellar basal body rod protein FlgG	2.4	down
Swit_1268	flagellar basal body FlaE domain-containing protein	2.3	down
Swit_1269	flagellar hook capping protein	2.1	down
Swit_1270	flagellar basal-body rod protein FlgC	3.3	down
Swit_1271	flagellar basal-body rod protein FlgB	2.3	down
Swit_1275	putative anti-sigma-28 factor, FlgM	3.0	down
Swit_1281	RNA polymerase, sigma 28 subunit, FliA/WhiG	2.3	down
Swit_1283	flagellin domain-containing protein	3.3	down
Swit_1284	flagellin domain-containing protein	2.6	down
Swit_1286	flagellar hook-basal body complex subunit FliE	4.4	down
Swit_1287	flagellar M-ring protein FliF	2.9	down
Swit_1293	flagellar basal body-associated protein FliL	3.8	down
Swit_1458	flagellar motor switch protein FliM	3.3	down

### Sodium chloride and PEG8000 have opposite effects on the degree of saturation of membrane fatty acids

**Figure 4:**
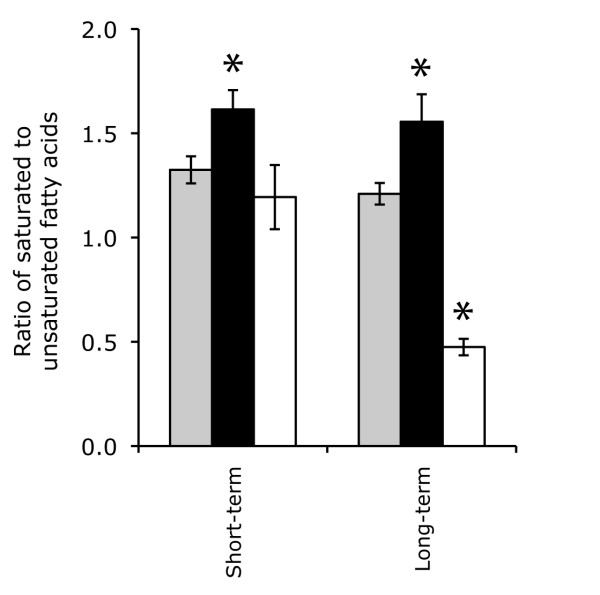
**The effect of sodium chloride or PEG8000 on the degree of saturation of membrane fatty acids**. The ratios of saturated to unsaturated fatty acids were measured in control cultures (grey bars), after perturbation with sodium chloride (black bars), or after perturbation with PEG8000 (white bars). Measurements were made after short-term perturbation (30 min) or long-term perturbation (24 hour). All measurements are averages from three biological cultures and error bars are one standard deviation. Asterisks (*) indicate measurements that are statistically different from the controls (*p*-value < 0.05).

FAME analyses were used to further investigate the responses to perturbation with sodium chloride or PEG8000 and to confirm that the applied perturbation levels led to physiological outputs. Short-term and long-term perturbation with sodium chloride significantly increased the ratio of saturated to unsaturated fatty acids when compared to the control (*p*-values < 0.05) (Figure 4). In contrast, short-term perturbation with PEG8000 had no significant effect on the ratio of saturated to unsaturated fatty acids (*p*-value > 0.05) while long-term perturbation with PEG8000 significantly decreased the ratio of saturated to unsaturated fatty acids (*p*-value < 0.05) (Figure 4). Thus, long-term perturbation with sodium chloride or PEG8000 had opposite effects on the degree of saturation of membrane fatty acids in strain RW1. These results were unexpected given that an increase in the degree of saturation of membrane fatty acids reduces the fluidity and permeability of the cell membrane and slows the rate of water loss in low water potential environments [49,50]. Thus, it was expected that perturbation with sodium chloride or PEG8000 would both lead to an increase in the degree of saturation of membrane fatty acids. Although it remains unclear why PEG8000 had the opposite effect than expected, the results provide physiological evidence that PEG8000 has a fundamentally different effect on the cytoplasmic membrane than sodium chloride and may even trigger antagonistic adaptive responses.

### Commonalities and differences between the responses to sodium chloride and PEG8000

Together, the data obtained in this investigation suggest the following hypothetical scenario for how strain RW1 responds to permeating and non-permeating solutes. After perturbation with the permeating solute sodium chloride, cells quickly begin to synthesize trehalose and exopolysaccharides, repair damaged proteins, and repress the synthesis of flagella. The cells also modify the composition of membrane fatty acids by increasing the degree of saturation. In the long-term, sodium chloride-perturbed cells return to their initial transcriptional state but maintain the increased degree of saturation of their membrane fatty acids. After perturbation with the non-permeating solute PEG8000, cells employ many of the same adaptive strategies used to respond to sodium chloride, including synthesizing trehalose and exopolysaccharides, repairing damaged proteins, and repressing the synthesis of flagella. However, cells up-regulate a broader range of heat shock-type chaperones and proteases, suggesting that PEG8000 damages cells in a fundamentally different way than sodium chloride. The cells also modify their membranes to decrease rather than increase the amount of saturated fatty acids. In the long-term, PEG8000-perturbed cells do not return to their initial transcriptional state and instead continue to repress flagella and pili biosynthesis. The differences in the responses to sodium chloride and PEG8000 may be partially controlled by different RNA polymerase sigma-factors, where ECF-type sigma 24 factors are up-regulated only after perturbation with sodium chloride while the heat shock-type sigma 32 factor is up-regulated only after perturbation with PEG8000.

## Conclusion

A combination of batch growth assays, transcriptome profiling, and membrane fatty acid analyses revealed that there is only a limited shared response to permeating and non-permeating solutes. Mostly, the responses to these solutes are different and might be under the control of different sigma factors. Thus, even though permeating and non-permeating solutes had the same effect on specific growth rates (Figure 1), these solutes affect cells in fundamentally different ways. Future work is now needed to test whether the responses to permeating and non-permeating solutes accurately simulate the responses to the solute and matric components of the total water potential, respectively, and to connect these responses with those observed in more realistic scenarios of soil desiccation.

## Authors' contributions

DRJ conceived the study, carried out the transcriptome profiling experiments, analyzed the transcriptome data, and drafted the manuscript. EC participated with the growth experiments. SKMF participated with the transcriptome profiling experiments. HH carried out the membrane fatty acid experiments and helped to draft the manuscript. JRM conceived the study and helped to draft the manuscript. All authors read and approved the final manuscript.

## Supplementary Material

Additional file 1 **Complete list of genes whose expression levels responded to short-term perturbation with sodium chloride or PEG8000 (FDR < 0.05, fold difference > 2.0)**. Click here for file

Additional file 2 **Complete list of genes whose expression levels responded to short-term perturbation with sodium chloride but not PEG8000 (FDR < 0.05, fold difference > 2.0)**. Click here for file

Additional file 3 Complete list of genes whose expression levels responded to short-term perturbation with PEG8000 but not sodium chloride (FDR < 0.05, fold difference > 2.0). Click here for file

Additional file 4 **Complete list of genes whose expression levels responded to long-term perturbation with PEG8000 (FDR < 0.05, fold difference > 2.0)**. Click here for file
